# Evaluation of a Multiplex Assay for Estimation of HIV-1 Incidence

**DOI:** 10.1371/journal.pone.0064201

**Published:** 2013-05-22

**Authors:** Kelly A. Curtis, Debra L. Hanson, M. Susan Kennedy, S. Michele Owen

**Affiliations:** Division of HIV/AIDS Prevention, National Center for HIV/AIDS, Hepatitis, STD, and TB Prevention, Centers for Disease Control and Prevention, Atlanta, Georgia, United States of America; Rush University, United States of America

## Abstract

**Objectives:**

Accurate methods of estimating HIV-1 incidence are critical for monitoring the status of the epidemic and the impact of prevention strategies. Although several laboratory-based tests have been developed strictly for this purpose, several limitations exist and improved methods or technologies are needed. We sought to further optimize a previously described bead-based, HIV-1-specific multiplex assay with the capability of measuring multiple immune responses for determining recent infection.

**Methods:**

We refined the customized HIV-1 Bio-Plex assay by determining cutoffs and mean durations of recency (MDR), based on the reactivity to longitudinal seroconversion specimens (n = 1347) from 311 ART-naïve, HIV-1-infected subjects. False-recent rates (FRRs) were calculated for various long-term cohorts, including AIDS patients, individuals on ART, and subtype C specimens. Incidence was estimated for each individual assay analyte from a simulated population with a known incidence of 1%. For improved incidence estimates, multi-analyte algorithms based on combinations of 3 to 6 analytes were evaluated and compared to the performance of each individual analyte.

**Results:**

The MDR for the six analytes varied from 164.2 to 279.4 days, while the multi-analyte algorithm MDRs were less variable with a minimum and maximum value of 228.4 and 277.9 days, respectively. The FRRs for the 7 multi-analyte algorithms evaluated in this study varied from 0.3% to 3.1%, in a population of ART-naïve, long-term individuals. All algorithms yielded improved incidence estimates as compared to the individual analytes, predicting an incidence of 0.95% to 1.02%.

**Conclusions:**

The HIV-specific multiplex assay described here measures several distinct immune responses in a single assay, allowing for the consideration of multi-analyte algorithms for improved HIV incidence estimates.

## Introduction

Controlling or eradicating the HIV epidemic remains a public health priority, as well as a major challenge. The estimation of new HIV infections in the population, or incidence, is crucial for understanding the status of the epidemic and providing information regarding the impact of prevention measures. In the last 15 years, several laboratory assays or tests for recent infection (TRIs) have been developed for distinguishing recent from long-term HIV infection, based on the reactivity to a specific biomarker [1]–[11]. These TRIs rely on the principle that reactivity to a particular biomarker, typically HIV-specific antibody levels or avidity, will increase predictably over time post-infection. An individual is classified as recently infected (i.e., infected within a defined mean duration of recency (MDR)) if the biomarker value is below the predetermined assay threshold. The BED-CEIA, which measures the proportion of IgG antibody directed towards an immunodominant gp41 oligopeptide, is the most well-known TRI and the first commercial assay designed specifically for the purpose of determining recent HIV infection [4], [12]. The BED assay has been employed worldwide for HIV incidence surveillance. To date, the HIV-1 Limiting Antigen (LAg)-Avidity EIA (Sedia Biosciences Corp., Portland, OR) is the only other TRI that has been commercialized for HIV-1 surveillance purposes. Like the BED assay, the LAg-Avidity EIA measures the reactivity to an antigen representing a subtype-conserved, immunodominant region of gp41; however, the antigen is “limited” on the assay plate to exclusively allow binding of high avidity antibodies [9], [10].

Recent concerns have been raised regarding the accuracy of current TRIs based on several reports describing the overestimation of HIV incidence in certain populations by the BED assay [13]–[15]. HIV-1 subtype diversity in the target population likely plays a role in the misclassification of long-term infections as recent or false-recent rate (FRR) associated with the BED assay, given that the MDR can vary from subtype to subtype [16]. Additionally, all serology-based TRIs are subject to some degree of error, as factors that disrupt the immune response to HIV, such as progression to AIDS and antiretroviral therapy (ART), will alter test performance; a phenomenon that has been well-documented [1], [3], [4], [11], [17]–[19]. All of these cofounding variables may contribute to inaccurate incidence estimates, which can have profound consequences for evaluating the impact of HIV prevention or surveillance program measures. Since these variables are present to some degree in most populations, along with innate immune variation, it is unlikely that an assay relying on a single biomarker value will reliably predict recent HIV infection in all settings.

Despite the challenges associated with current TRIs, laboratory-based assays remain attractive for incidence estimation because they are relatively easy to perform on cross-sectional samples and are less costly than cohort studies, which involve regular monitoring of high-risk HIV-negative individuals in order to identify new infections. Given these advantages, there is a pressing need to improve the performance of current TRIs or to identify novel biomarkers and/or technologies that compensate for immune diversity, thereby yielding low FRRs (≤2%) and acceptable MDRs (between 4 and 12 months) [20]. In August of 2011, the WHO Working Group on HIV Incidence Assays was convened to discuss the relevance of new and existing TRIs and to develop guidelines for those seeking to design new assays [21]. One approach gaining consideration is the use of an algorithm based on multiple tests and/or clinical data to improve the accuracy of incidence estimates. Recent studies examining the utility of an algorithm based on multiple TRIs in conjunction with clinical data, such as CD4 counts, have shown improved specificity as compared to each individual test [22], [23]. The algorithm approach, however, is not without its potential logistical problems, since the requirement for multiple tests would be prohibitively expensive and difficult to implement in certain testing settings.

Recently, we described the development of a bead-based multiplex assay for determining recent HIV-1 infection, using the Bio-Plex platform [24]. While most TRIs measure HIV-specific antibody levels or avidity, the customized Bio-Plex assay incorporates both measures against multiple analytes in a single assay format. Preliminary analyses indicated a measurable difference in reactivity between specimens of known recent and long-term infection for seven different analytes. Here, we describe the further refinement of the assay, including estimation of cutoff values, corresponding MDRs, and FRRs for each individual analyte in the assay. We also evaluate the utility of multi-analyte algorithms based on different combinations of analytes for minimizing FRRs and improving incidence estimation.

## Materials and Methods

### Longitudinal Seroconversion Panels and Cohorts

For estimation of cutoff values, MDR, and incidence, 1347 specimens collected from 311 ART-naïve, HIV-1-infected subjects were evaluated. Longitudinal seroconversion specimens from subtype B HIV-infected individuals were obtained commercially or from several prospective studies and described as follows: 12 HIV-1 seroconversion panels (n = 62) were purchased from Zeptometrix Corp. (Buffalo, NY) and 14 panels (n = 42) were obtained from SeraCare Life Sciences (formerly Boston Biomedica Inc.; Milford, MA). Longitudinal specimens from 95 recent seroconverters (n = 397) were collected as part of the Vaccine Preparedness Study for the HIV Network for Prevention Trials (HIVNET; ClinicalTrials.gov identifier NCT00000915), as described in detail [24]–[26]. For this particular cohort, the interval of time between the last negative and first positive antibody test varied greatly, from 30–364 days. Specimens from 62 subjects (n = 274) were obtained from the AIDSVAX B/B Phase III Vaccine Trial (VAX004; ClinicalTrials.gov identifier NCT00002441) [27]. The double-blind, randomized trial involved the evaluation of a candidate vaccine composed of recombinant gp120 antigens (AIDSVAX B/B; VaxGen Inc, South San Francisco, CA). Study participants were enrolled at various sites in North America and The Netherlands and included men who have sex with men (MSM) and high-risk heterosexual women. To avoid confounding variables elicited from the vaccine, only specimens from placebo recipients that became HIV infected during the vaccine trial were evaluated in the present study. Once an individual became infected, samples were collected at <1 month and 1, 2, 4, 8, 12, 16, 20, and 24 months post-diagnosis.

Longitudinal seroconversion specimens from non-B subtype infections were obtained from two separate studies. Specimens from 105 subjects (n = 349) with HIV-1 subtype B and E infections were collected as part of the AIDSVAX B/E Phase III Vaccine Trial (VAX003; ClinicalTrials.gov identifier NCT00006327), evaluating a bivalent recombinant gp120 protein among injection drug users (IDUs) in Bangkok, Thailand [28], [29]. As described for VAX004, only specimens from study subjects that became infected while receiving the placebo were included in our evaluations. Additionally, 14 seroconverters (n = 131) of subtypes G and A/G were identified through the Recruiting Acute Cases of HIV (REACH) study. Acute cases of HIV-1 were obtained from screening high-risk individuals in Abuja and Jos, Nigeria. Study designs and sample collection have been described in detail previously [24], [30].

### Specimens from Long-term Infections

For estimation of false-recent rate (FRR), samples from known long-term infected individuals (collected >365 days post-seroconversion) were evaluated. Longitudinal specimens (n = 708) from 103 subjects were obtained from a prospective study involving HIV-1 infected MSM diagnosed with unexplained, generalized lymphadenopathy syndrome [31]–[33]. Study participants were enrolled between 1982 and 1983 in Atlanta, Georgia and monitored at 3- to 6-month intervals for clinical and immunological evaluation of progression to AIDS, including CD4+ T cell count determination. Since last negative and first positive antibody test dates are unavailable for this cohort, samples were excluded if the sample collection date was <365 days from study entry or initial sample collection. Of the 103 subjects evaluated in the present study, 47 eventually progressed to AIDS, as determined by CD4+ T cell count.

The impact of ART treatment and subtype diversity on FRR was evaluated using the following cohorts: 67 subjects (n = 393 specimens) from the HIVNET cohort received ART at the time of one or more sample collections. An estimated time from seroconversion to ART initiation was determined based upon the ART status of the study subject at the time of each sample collection. A collection of subtype C specimens (n = 67) from ART-naïve long-term individuals (CHAVI001) was obtained through the Center for HIV/AIDS Vaccine Immunology [34].

### HIV-1 Multiplex Assay

The HIV-1-specific Bio-Plex assay was performed as previously described [24] for the detection of IgG reactivity and avidity directed against COOH microspheres (Bio-Rad Laboratories, Hercules, CA) coupled with the following recombinant HIV-1 proteins: p66 (Protein Sciences Corp., Meriden, CT), gp120, gp160, and gp41 (Immunodiagnostics, Inc., Woburn, MA). All plasma/serum samples were tested in duplicate under both treatment conditions, with and without diethylamine (DEA). A normalized mean fluorescent intensity (MFI) value and avidity index were calculated as previously described [24].

### Determination of Cutoff Values

Cutoff values for recent/long-term classification were determined as described previously [24] for the following analytes: gp120-normalized MFI value (n), gp160-n, p66-avidity index (a), gp120-a, gp160-a, and gp41-a. Based on the large degree of overlap in reactivity to p66-n between known recent and long-term specimens [24], this analyte was not included in the current study. Normalized values for gp120 and gp160 were fit to 2-parameter nonlinear regression models [35] with random effects. The model formula is Y = (x*A)/(B+x), where Y is analyte response at x days since seroconversion, A is the maximum value of the analyte response, and B is the time since seroconversion at half maximal response. In addition to visualizing how well the model curve approximates the true curve, goodness of fit was assessed using residual plots and residual variance. For the purposes of curve-fitting, time since seroconversion was defined as the midpoint between the last negative and first positive Western Blot result. The intervals of time between the last negative and first positive test date did not exceed 365 days, with a minimum of 1 day and a maximum of 364 days.

Subject-specific avidity measurements were fit to a 4-parameter logistic (4 PL) nonlinear regression model [36], with a random effect to account for the within-subject correlation of measurements [37], [38]. The 4 PL model equation is Y = D+(A−D)/(1+(x/C)^ B^), where Y is analyte response (avidity index) at time (x days) since seroconversion, A is the background level (lower limit) analyte response, D is the maximum level (upper limit) analyte response, C is the mid-range inflection point on the curve, and B is a slope factor or steepness of the curve. This function provides an accurate representation of the sigmoidal relationship between the measured response and time since seroconversion.

Selection of a cutoff value for defining recent infection was characterized by analyte values that are as high as possible but no greater than the model-predicted half maximal response, i.e. analyte values at which the slope begins to decrease and analyte values are leveling. The half maximal response or inflection point is a well-characterized parameter of fitted regression curves, which is reached within the initial year post-seroconversion for the Bio-Plex analytes. Therefore, we defined a plausible cutoff value that was between the estimated half maximal response and the lower 99% confidence limit of this estimate.

### Selection of Analyte Combinations and Cutoff Criteria

To evaluate the performance of multi-analyte algorithms, various combinations of 3–6 analytes were selected. Since antibody avidity appears to be a robust predictor of recent infection, 3–4 avidity measures were included in each algorithm, with or without the least predictive analyte, p66-a. In addition to the avidity measures, one or both of the normalized values, gp120-n and gp160-n, were included in all but one of the algorithms. The cutoff criteria for each algorithm, as listed in Table 1, indicate the number of analytes, out of the total included in the algorithm, that must cross the threshold established for each analyte (recent/long-term cutoff for each analyte) in order to classify the individual as having progressed from recent to long-term infection. For example, a cutoff criterion of 3/5 indicates that a particular individual is considered recent until 3 out of the 5 analytes in the combination yields values above their respective cutoffs. Multiple cutoff criteria were evaluated for each algorithm, however, only those that provided the best incidence estimates are shown in Table 1.

**Table 1:** Characterization of the HIV-1-specific Bio-Plex.

Analyte/Algorithm	Cutoff	MDR	FRR	Incidence (95% CI)	% Difference^b^
gp160-a	25	235.1	1.1	1.07 (1.04, 1.10)	7.2
gp120-a	20	265.0	4.5	1.07 (1.04, 1.10)	6.6
gp41-a	35	223.6	3.4	1.11 (1.08, 1.15)	11.5
p66-a	10	279.4	27.8	1.14 (1.08, 1.20)	14.2
gp160-n	5	164.2	0.3	1.18 (1.14, 1.22)	18.1
gp120-n	7	175.9	8.4	1.19 (1.13, 1.26)	19.5
160 n, 120 n, 66a, 120a, 160a, 41a	3/6^a^	228.4	0.3	1.02 (1.00, 1.05)	2.4
160 n, 66a, 120a, 160a, 41a	3/5	256.6	1.1	.98 (0.95, 1.00)	−2.5
120 n, 66a, 120a, 160a, 41a	3/5	264.2	2.3	.95 (0.93, 0.98)	−4.8
160 n, 120n, 120a, 160a, 41a	3/5	238.6	0.3	1.02 (1.00, 1.04)	2.0
120 n, 120a, 160a, 41a	3/4	277.9	3.1	0.99 (0.96, 1.01)	−1.4
160 n, 120a, 160a, 41a	3/4	265.6	1.4	1.00 (0.98, 1.03)	0.4
120a, 160a, 41a	2/3	250.3	1.4	1.02 (0.99, 1.05)	2.0

a Algorithm cutoffs are listed as the number of analytes that must measure above each analyte-specific cutoff to be considered long-term over the total number of analytes in the algorithm.

b Relative % difference from actual population incidence.

### Estimation of Mean Duration of Recency (MDR)

The MDR was estimated for each individual analyte and for each analyte combination or algorithm. Survival methods used to estimate the MDR between time of seroconversion and time when a selected biomarker cutoff value is reached have been described previously [16]. Briefly, time since seroconversion was multiply imputed 10 times using a predictive mean matching regression method for data with monotone missing patterns [39], conditional on its occurrence between the last negative and first positive HIV test dates. Before application of the imputation model, linear mixed effects regression of days since the midpoint between last negative and first positive HIV tests was performed on analyte measurements to estimate the increase per day (slope) and model intercept. In addition to slope and intercept, the imputation model included covariates for HIV subtype, time since seroconversion, and analyte measurement value for the observation with the closest fit to the estimated linear regression slope. We assumed the seroconversion dates were non-missing and equal to the midpoint of the interval for those seroconverters with intervals of ≤90 days between last negative and first positive tests. For seroconverters with at least one measurement greater than the selected cutoff value, the estimated time when the cutoff was reached was linearly interpolated from times at which values first reached the selected cutoff value and the preceding longitudinal observation. Observations were right-censored at the time of the highest value for those seroconverters who did not have a value greater than the selected cutoff. The MDR for a predefined combination of 3–6 analytes is the estimated time between seroconversion and when the selected cutoff value for the 2^nd^ or 3^rd^, depending on the criterion, analyte is reached.

A recently published, improved estimator for HIV incidence introduces a timescale, T, describing the dynamic range of recency [40]. The MDR, or average time spent alive and recently infected at T = 1 year, is calculated using the trapezoidal rule for estimation of area under the curve [41], [42].

### Estimation of False-recent Rate (FRR)

The second important parameter for estimation of HIV incidence, the FRR, is the probability that a person infected for longer than T will be misclassified as recently infected by having a measurement value below the selected cutoff value for a given analyte. FRR was calculated from specimens collected from persons with known long-term infection (>365 days post-seroconversion). Exact binomial confidence limits (95%) were calculated.

### Estimation of Incidence

For an overall evaluation of the analytes, singly or in combination, the two calculated incidence parameters, MDR & FRR, were used to calculate incidence (I) in a simulated population using the following formula: I = (R – FRR*P)/(N*(MDR-FRR*T)), where R is the number of recent infections, P is the total number of prevalent infections, N is the total number of HIV-negatives, and FRR, MDR, and T are as previously defined. For modeling incidence estimation, data were randomly split into two datasets. Although the collective data were obtained from the same cohorts, one set of data was used to calculate the MDR and FRR parameters; the other set was used for estimation of incidence. To better reflect cross-sectional data, a bootstrap resampling (100 replicates) of the incidence dataset was performed such that the data were uniformly distributed with respect to time since seroconversion within two time frames,<and ≥ T = 1 year. In addition, data <T were sampled to represent 40% of the data set. The total number of HIV-negative (N) entered into the incidence formula was fixed at a number such that the true incidence was 1%. For example, 1825 prevalent HIV infections were randomly selected within uniform distribution in both time periods. Of these, 730 were recent and 1095 were long-term (40% of all HIV infections are recent). For 1% incidence, given 730 recent infections per 73000 negative or recent, the total number of HIV negative is 72270. In this example, R = 730, P = 1825, N = 72270, where R and P reflect bootstrap resampling from the actual data with constraints for their uniform distribution over time. Relative percent difference from 1% incidence was calculated based upon our results from the Bio-Plex analytes or combinations of analytes.

## Results

### Cutoff Values and Mean Duration of Recency

**Figure 1:**
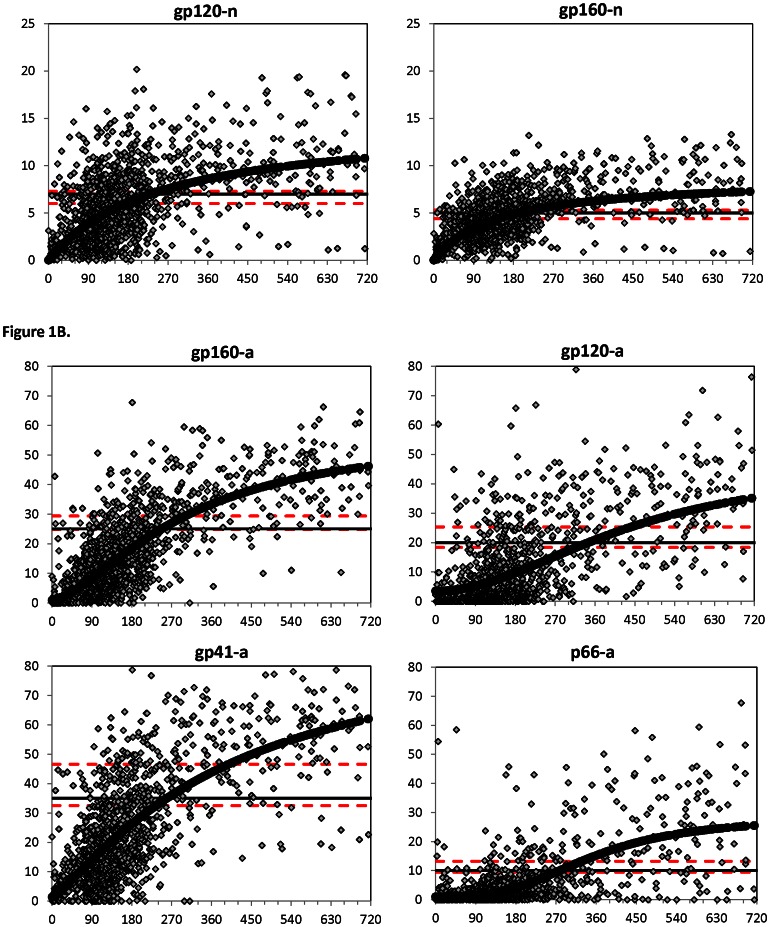
Longitudinal HIV-1-specific antibody responses. The normalized values (A) and avidity index (B), as measured by the Bio-Plex assay, for longitudinal seroconverters were plotted over days since seroconversion. Gray diamonds indicate individual data points and solid black circles represent the curve fit for each graph. Dashed red lines represent the half maximal response of the curve and lower 99.9% confidence limits of the estimate, while the solid black line represents the selected cutoff.

Antibody binding and avidity to the analytes, gp120-n, gp160-n, p66-a, gp120-a, gp160-a, and gp41-a, were measured on the Bio-Plex assay for a total of 1347 specimens. For all analytes, a similar increase in reactivity post-seroconversion was observed; however, the range of reactivity and maximum attainable values varied from analyte to analyte (Figure 1). Selected cutoff values for each analyte are displayed in the graphs as solid black lines and were chosen based on the inflection point in the curve fit. The cutoff values for each individual analyte and combination of analytes that were selected for further evaluation are listed in Table 1.

The MDR for the six analytes varied from 164.2 to 279.4 days (Table 1), reflecting the differing kinetics of antibody reactivity shown in Figure 1. The mean interval for a seroconverter to reach the analyte-specific cutoff was shorter for the normalized values, as compared to the avidity measures (Table 1). The median MDR for the individual analytes was 229.4 days. Algorithm MDRs ranged from 228.4 to 277.9 days, with a median MDR of 256.6 days.

### Estimation of HIV Incidence

To evaluate the performance of the Bio-Plex assay in estimating incidence, the FRRs of representative long-term populations were calculated for each analyte and algorithm (Table 1). The FRR varied considerably between the analytes, ranging from 0.3–27.8%. The gp160 protein was associated with the lowest misclassification rate, yielding FRRs of 0.3% and 1.1% for the normalized values and avidity index, respectively. In contrast, p66-a was the least specific analyte, with a FRR of 27.8%. The FRRs for the 7 algorithms were considerably less variable, with a minimum and maximum value of 0.3% and 3.1%, respectively.

HIV-1 incidence was calculated based on a resampled data set with a known incidence of 1% (Table 1). All of the individual analytes overestimated incidence, with estimates ranging from 1.07% to 1.19%. The relative difference, as compared to actual incidence, ranged from 7.2% to 19.5%. The multi-analyte algorithms improved incidence estimates in all cases. The algorithm estimates ranged from 0.95% to 1.02%, with relative differences from actual incidence ranging from -4.8% to 2.4%. The 95% confidence intervals included the expected incidence of 1% for all but one algorithm.

### False-recent Rate in Challenging Cohorts

The value of a multi-analyte algorithm compared to a single assay measure was evaluated by comparing the FRR in cohorts typically associated with higher misclassification rates (Table 2). To determine the potential impact of AIDS progression on the FRR, values were compared for a long-term MSM cohort with specimens collected prior to and after progression to AIDS. The FRRs were similar regardless of whether the AIDS specimens were included in the estimates, exhibiting a minimum and maximum value for the individual analytes of 0.7% and 28.2% with AIDS cases and 0.6% and 27.8% for non-AIDS cases (Table 2). In both groups, the gp160 antigen elicited the lowest FRRs (≤1%). As compared to the values for the individual analytes, the lowest FRR of the multi-analyte algorithms dropped to 0.1% and 0.2% for the MSM cohort with and without AIDS, respectively.

**Table 2:** Impact of disease progression and antiretroviral use on false-recent rates.

	FRR (95% CI)				
	MSM no AIDS	MSM+AIDSa	HIVNET no ART	HIVNET ART-early initiation	HIVNET ART-late initiation
Analyte/Algorithm	(n = 540)	(n = 708)	(n = 138)	(n = 299)	(n = 94)
gp160-a	0.7 (0.2, 1.9)	0.7 (0.2, 1.6)	10.9 (6.2, 17.3)	16.4 (12.4, 21.1)	3.2 (0.7, 9.0)
gp120-a	2.6 (1.4, 4.3)	3.2 (2.1, 4.8)	11.6 (6.8, 18.1)	7.0 (4.4, 10.5)	13.8 (7.6, 22.5)
gp41-a	2.8 (1.6, 4.5)	2.4 (1.4, 3.8)	9.4 (5.1, 15.6)	24.7 (20.0, 30.0)	11.7 (6.0, 20.0)
p66-a	27.8 (24.0, 31.8)	28.2 (25.0, 31.7)	27.5 (20.3, 35.8)	39.8 (34.2, 45.6)	44.7 (34.4, 55.3)
gp160-n	0.6 (0.1, 1.6)	1.0 (0.4, 2.0)	7.2 (3.5, 12.9)	26.1 (21.2, 31.5)	6.4 (2.4, 13.4)
gp120-n	3.5 (2.1, 5.4)	7.5 (5.7, 9.7)	8.7 (4.6, 14.7)	36.1 (30.7, 41.9)	28.7 (19.9, 39.0)
160n, 120n, 66a, 120a, 160a, 41a	0.2 (0.0, 1.0)	0.1 (0.0, 0.8)	4.3 (1.6, 9.2)	17.7 (13.6, 22.5)	4.3 (1.2, 10.5)
160n, 66a, 120a, 160a, 41a	0.9 (0.3, 2.2)	0.7 (0.2, 1.6)	5.1 (2.1, 10.2)	18.1 (13.9, 22.9)	4.3 (1.2, 10.5)
120n, 66a, 120a, 160a, 41a	1.1 (0.4, 2.4)	1.3 (0.6, 2.4)	5.1 (2.1, 10.2)	18.4 (14.2, 23.3)	9.6 (4.5, 17.4)
160n, 120n, 120a, 160a, 41a	0.4 (0.0, 1.3)	0.6 (0.2, 1.4)	7.2 (3.5, 12.9)	19.7 (15.4, 24.7)	4.3 (1.2, 10.5)
120n, 120a, 160a, 41a	1.3 (0.5, 2.7)	2.0 (1.1, 3.3)	9.4 (5.1, 15.6)	21.4 (16.9, 26.5)	12.8 (6.8, 21.2)
160n, 120a, 160a, 41a	0.9 (0.3, 2.2)	1.1 (0.5, 2.2)	9.4 (5.1, 15.6)	20.1 (15.7, 25.1)	5.3 (1.8, 12.0)
120a, 160a, 41a	0.9 (0.3, 2.2)	0.8 (0.3, 1.8)	6.5 (3.0, 12.0)	14.0 (10.3, 18.5)	4.3 (1.2, 10.5)

a Includes entire MSM cohort.

Since ART can confound serologic-based TRIs [17]–[19], we evaluated long-term specimens from a longitudinal cohort with known dates of ART initiation. For baseline comparison, we first evaluated samples that were collected from ART-naïve individuals or at time points prior to ART initiation. In general, the HIVNET cohort was associated with a high FRR, as all analytes and algorithms exhibited FRRs greater than 2%. To determine the impact of ART treatment on the performance of the multi-analyte assay, the ART-treated specimens were designated as “early” or “late”, based on whether ART was initiated within 365 days post-seroconversion or after. Early ART initiation was associated with a higher FRR, as compared to the ART-negative population (Table 2). However, when ART was initiated greater than a year post-seroconversion, the FRRs for most of the individual analytes and algorithms were similar to the ART-negative group. In the absence of ART, four of the algorithms exhibited lower FRRs as compared to the best performing individual analyte, gp160-n. In general, the FRRs were lower for the algorithms as compared to the individual analytes; however, the added advantage of multiple analytes was less powerful when ART was initiated within the first year post-seroconversion. With the exception of the early ART treatment group, the six-analyte algorithm provided the lowest FRR of all algorithms evaluated in this study (Table 2).

Lastly, the performance of the Bio-Plex assay on one non-B subtype specimen set was assessed using a cohort of subtype C specimens from ART-naïve long-term individuals. Gp160-a, gp120-a, and all seven of the algorithms exhibited a FRR of 0% (Table 3).

**Table 3:** Bio-Plex assay performance on subtype C specimens.

	FRR (95% CI)
Analyte/Algorithm	CHAVI (n = 67)
gp160-a	0.0 (0.0, 5.4)
gp120-a	0.0 (0.0, 5.4)
gp41-a	1.5 (0.0, 8.0)
p66-a	4.5 (0.9, 12.5)
gp160-n	1.5 (0.0, 8.0)
gp120-n	1.5 (0.0, 8.0)
160n, 120n, 66a, 120a, 160a, 41a	0.0 (0.0, 5.4)
160n, 66a, 120a, 160a, 41a	0.0 (0.0, 5.4)
120n, 66a, 120a, 160a, 41a	0.0 (0.0, 5.4)
160n, 120n, 120a, 160a, 41a	0.0 (0.0, 5.4)
120n, 120a, 160a, 41a	0.0 (0.0, 5.4)
160n, 120a, 160a, 41a	0.0 (0.0, 5.4)
120a, 160a, 41a	0.0 (0.0, 5.4)

## Discussion

In this study, we evaluated the utility of multi-analyte algorithms for improved HIV incidence estimates using the Bio-Plex assay. Although the HIV-specific antibody responses displayed similar patterns of reactivity, each analyte exhibited a unique rate of increase and scale of reactivity. It was, therefore, necessary to consider each analyte as a separate test, with a distinct cutoff and MDR estimate. Our preliminary analyses indicated that selecting analyte cutoffs based on the natural inflection point in the curve of longitudinal reactivity allowed for the greatest distinction in reactivity between recent and long-term specimens with the Bio-Plex assay, as opposed to selecting the cutoff based on a set MDR (e.g., 180 or 365 days; data not shown). When relying on a single-assay measure, a certain percentage of the population may never reach the threshold for a particular biomarker due to innate differences in the immune response from individual to individual. Therefore, cutoff criteria for the analyte combinations or algorithms were selected to allow for some degree of immune variation. Instead of basing the classification of recent infection on the full agreement of each test or analyte result, a pre-determined number of analytes less than the total included in the algorithm needed to meet the criteria or score above the threshold to consider an individual long-term. Based on the algorithm cutoff criteria described here, one or more analytes can “fail” or never reach the analyte cutoff, without affecting the final assay determination for an individual infected for a time period longer than the MDR. A minimum of three analytes were included in each algorithm to maintain the described cutoff criteria.

Since it was unknown whether an algorithm based on TRIs or assay measures would improve HIV incidence estimates, we evaluated the performance of each individual analyte in comparison to various multi-analyte algorithms. One of the biggest challenges in validating candidate TRIs is the difficulty in obtaining well-characterized cohorts with known incidence rates. For proof-of-concept purposes, we used resampled data to generate a simulated ART-naïve cohort with a known incidence of 1%. A limitation of the current study is that the simulated population for incidence evaluation was derived from the same cohorts used to calculate the MDRs and FRRs for the individual analytes and algorithms. Even though all of the algorithms, regardless of whether 3, 4, 5, or 6 analytes were included, yielded improved incidence estimates as compared to the individual analytes, it is essential to validate algorithm performance in diverse populations, especially those associated with high FRRs. Reasons for high FRRs in specific populations may be numerous, so we assessed two confounding factors that are typically associated with a reduction in the antibody response to HIV, progression to AIDS and ART. Individuals that progress to AIDS often exhibit a decline in HIV-specific antibody levels, leading to a higher likelihood of misclassification by some TRIs [1], [3], [4], [11]. In contrast, we observed minimal to no impact on antibody reactivity when specimens from AIDS cases were included in the analyses, which is likely due to the high sensitivity of the Bio-Plex assay format. ART use is also a well-documented challenge for most antibody-based assays, given that reduction in viral loads leads to reduced antigenic stimulation necessary for antibody production and maturation. In this study, we observed a notable difference in assay performance depending on the timing of ART initiation post-seroconversion, which indicated that a sustainable HIV-specific antibody response is dependent upon adequate virus replication within the first year post-infection.

In general, one or more of the analyte algorithms exhibited lower FRRs as compared to the individual analytes; however, the added advantage of multiple analytes was less convincing for the early ART initiation group which had unusually high FRRs. For the early ART specimens, one individual analyte (gp120-a) exhibited a FRR lower than all of the algorithms, including non-overlapping 95% confidence limits. It is not known whether this finding is meaningful or the result of small sample sizes or other cohort-specific variables. Although the impact of early ART on assay performance is clear, the cohort used in this study was associated with a high FRR in general, as also demonstrated by the BED assay (FRR = 10%; data not shown). It is likely that the high FRR associated with this particular cohort is due to relatively large intervals of time between the last negative and first positive antibody test dates for the majority of the study participants, leading to uncertainty around the estimated seroconversion dates. Duration of time on ART may also correlate with an increase in misclassification; however, this variable was not addressed in the present study. Additionally, viral load data were not available for this cohort, so the direct effect of ART-induced virus suppression on assay performance could not be measured. Further investigation, using well-characterized longitudinal specimens with relatively short sample collection intervals, is needed to fully assess the potential benefit of a multi-analyte algorithm in populations with high ART use. Furthermore, ongoing research involves the evaluation of additional biomarkers that may not be affected by declining virus levels. One such candidate is anti-p24 IgG_3,_ which has been shown to peak during early infection, but unlike IgG_1_ levels, declines to low or undetectable levels shortly thereafter [43]. Preliminary studies in our laboratory have indicated that peak IgG_3_ levels occur prior to the initiation of ART in most individuals (data not shown). Moreover, the Bio-Plex format is not limited to the detection of antibody biomarkers, such that alternative immune activation biomarkers can be assessed.

The assay performance described here is highly encouraging; however, all new incidence assays or technologies must be carefully validated prior to field implementation. The current assay format will be further assessed to determine inter-lab variation and performance on diverse HIV-1 subtypes. Since the Bio-Plex assay was designed with subtype B antigens, care must be taken to avoid generalizing about assay performance with diverse subtypes based on the reactivity of subtype B samples. We observed a low or zero FRR with subtype C samples from long-term individuals, however, the cohort size was relatively small and additional subtypes were not evaluated due to lack of availability. To address potential discrepancies in the reaction kinetics of non-B subtype specimens, we plan to evaluate subtype conserved peptides and recombinant proteins, in addition to the current analytes.

One challenge that we expect to face in validating the multiplex assay is identifying criteria for selecting the best algorithm or algorithms for use in diverse populations. Although FRR is a valuable measurement for test evaluation, it does not necessarily predict the analytes or algorithms that provide the most accurate incidence estimates. However, since FRR is a key component of the formula used to calculate HIV incidence, it is desirable to identify a combination of analytes that produces the lowest or negligible FRR, while maintaining a sufficient MDR for feasible incidence calculations. Further testing is also needed to determine the necessity of each recombinant protein included in the current format of the assay. Another potential challenge of the current assay format is the need for dedicated equipment that may be difficult to acquire in certain testing settings. Future plans include the identification and evaluation of portable versions of the Bio-Plex format and/or alternative, low-tech platforms with multiplexing capability.

The results described here demonstrate the advantage of a multiplex system that enables measurement of multiple analytes for improved cross-sectional HIV-1 incidence estimates. We demonstrate that a multi-analyte algorithm based on three or more assay measures provides lower FRRs and improved incidence estimates. We emphasize that all cutoffs and MDRs were estimated for proof-of-concept evaluation only and may change after further refinement of the assay.
